# Supplementary material for: Investigating the influence of physiologically relevant hydrostatic pressure on CHO cell batch culture
Source: Sci Rep. 2021 Jan 8;11:162. doi: 10.1038/s41598-020-80576-8 (PMC7794228; doi:10.1038/s41598-020-80576-8)
Supplement: Supplementary file 1 — Supplementary Information 1. [file 41598_2020_80576_MOESM1_ESM.zip › S1/FLEX2/13Jan 30mmHg SS1 is C/Blank 2.pdf]

===

Acq. Operator : SYSTEM Seq. Line : 3
Sample Operator : SYSTEM
Acq. Instrument : HPLC-0039 Location : 1
Injection Date : 1/14/2019 3:34:19 PM Inj : 1
Inj Volume : 100.000 µl
Sequence File : C:\Users\Public\Documents\ChemStation\1\Data\TK_CHO_IgG 2019-01-14 15-24-10\TK_CHO_IgG.S
Method : C:\Users\Public\Documents\ChemStation\1\Data\TK_CHO_IgG 2019-01-14 15-24-10\CHO_IgG_LC.M (Sequence Method)
Last changed : 12/4/2018 1:38:41 PM by SYSTEM

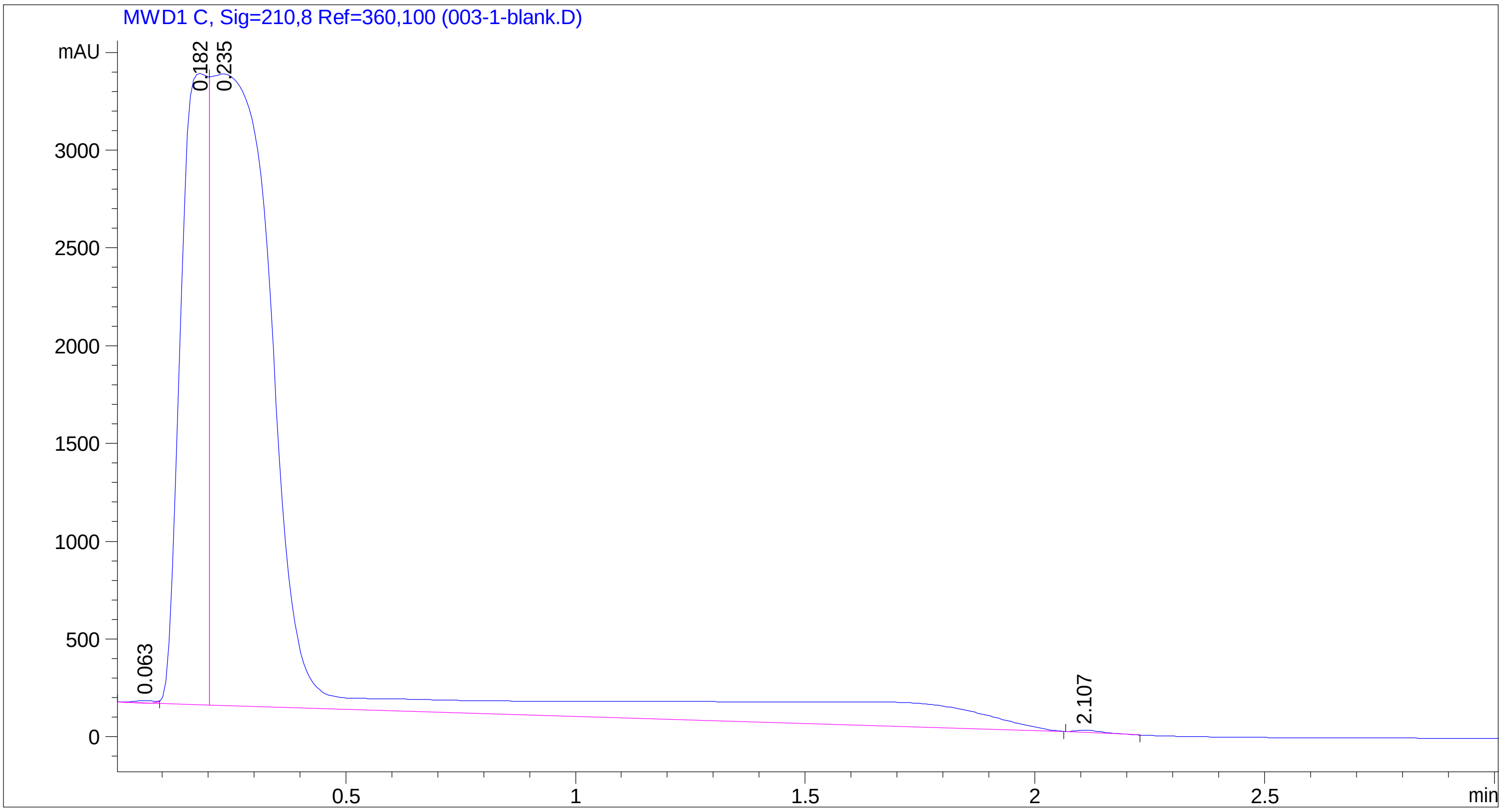

===

Area Percent Report

===

Sorted By : Signal
Multiplier : 1.0000
Dilution : 1.0000
Use Multiplier & Dilution Factor with ISTDs

Signal 1: MWD1 C, Sig=210,8 Ref=360,100

Peak #	RetTime [min]	Type	Width [min]	Area [mAU*s]	Height [mAU]	Area %
1	0.063	BV E	0.0440	38.03369	13.06573	0.0770
2	0.182	VV R	0.0636	1.31681e4	3227.59106	26.6576
3	0.235	VB	0.1666	3.61478e4	3229.81030	73.1781
4	2.107	BBA	0.0625	43.13348	10.81229	0.0873

Totals : 4.93971e4 6481.27939

===

*** End of Report ***
